# Pricing and cost-saving potential for deep-learning computer-aided lung nodule detection software in CT lung cancer screening

**DOI:** 10.1186/s13244-023-01561-z

**Published:** 2023-11-27

**Authors:** Yihui Du, Marcel J. W. Greuter, Mathias W. Prokop, Geertruida H. de Bock

**Affiliations:** 1 Department of Epidemiology and Health Statistics, School of Public Health, Hangzhou Normal University, Hangzhou, China; 2 Department of Epidemiology, University Medical Center Groningen, University of Groningen, Groningen, The Netherlands; 3 Department of Radiology, University Medical Center Groningen, University of Groningen, Groningen, The Netherlands; 4 Department of Medical Imaging, Radboud University Medical Center, Nijmegen, The Netherlands

**Keywords:** Deep learning, Computed aid detection, Pricing, Lung nodule, Lung cancer screening

## Abstract

**Objective:**

An increasing number of commercial deep learning computer-aided detection (DL-CAD) systems are available but their cost-saving potential is largely unknown. This study aimed to gain insight into appropriate pricing for DL-CAD in different reading modes to be cost-saving and to determine the potentially most cost-effective reading mode for lung cancer screening.

**Methods:**

In three representative settings, DL-CAD was evaluated as a concurrent, pre-screening, and second reader. Scoping review was performed to estimate radiologist reading time with and without DL-CAD. Hourly cost of radiologist time was collected for the USA (€196), UK (€127), and Poland (€45), and monetary equivalence of saved time was calculated. The minimum number of screening CTs to reach break-even was calculated for one-time investment of €51,616 for DL-CAD.

**Results:**

Mean reading time was 162 (95% CI: 111–212) seconds per case without DL-CAD, which decreased by 77 (95% CI: 47–107) and 104 (95% CI: 71–136) seconds for DL-CAD as concurrent and pre-screening reader, respectively, and increased by 33–41 s for DL-CAD as second reader. This translates into €1.0–4.3 per-case cost for concurrent reading and €0.8–5.7 for pre-screening reading in the USA, UK, and Poland. To achieve break-even with a one-time investment, the minimum number of CT scans was 12,300–53,600 for concurrent reader, and 9400–65,000 for pre-screening reader in the three countries.

**Conclusions:**

Given current pricing, DL-CAD must be priced substantially below €6 in a pay-per-case setting or used in a high-workload environment to reach break-even in lung cancer screening. DL-CAD as pre-screening reader shows the largest potential to be cost-saving.

**Critical relevance statement:**

Deep-learning computer-aided lung nodule detection (DL-CAD) software must be priced substantially below 6 euro in a pay-per-case setting or must be used in high-workload environments with one-time investment in order to achieve break-even. DL-CAD as a pre-screening reader has the greatest cost savings potential.

**Key points:**

• DL-CAD must be substantially below €6 in a pay-per-case setting to reach break-even.

• DL-CAD must be used in a high-workload screening environment to achieve break-even.

• DL-CAD as a pre-screening reader shows the largest potential to be cost-saving.

**Graphical Abstract:**

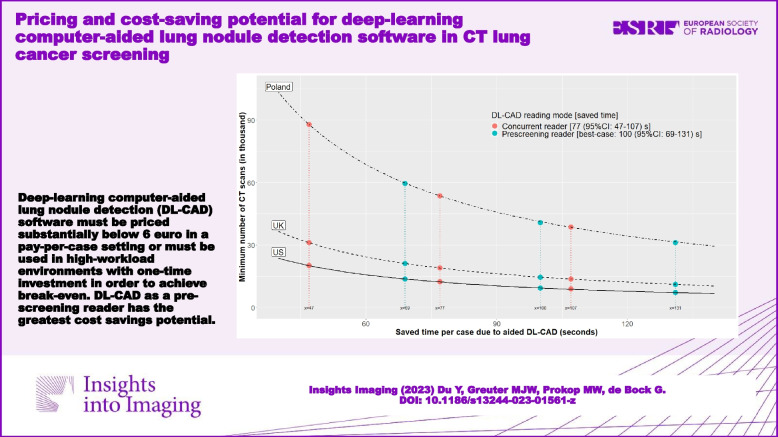

**Supplementary Information:**

The online version contains supplementary material available at 10.1186/s13244-023-01561-z.

## Introduction

Many business cases involving the use of artificial intelligence (AI) for reporting of radiologic studies aim at proving savings in the value chain downstream from radiology reporting, trying to quantify the benefit of better diagnoses. In these use cases, there is often little immediate benefit for radiology departments that would allow financing of these software solutions. Immediate benefits, however, occur if the software improves reporting workflow and increases reporting efficiency, which could provide a business case for the AI solution within a radiology practice. Our study explores this idea for the example of deep learning computer-aided detection (DL-CAD) systems for detection of pulmonary nodules in a computed tomography (CT) lung cancer screening setting. We chose this example because nodule CAD is a popular application of AI for which a large and growing number of commercial nodule CAD solutions are available [1]. In addition, a lung screening setting provides an environment in which large numbers of cases may need to be evaluated.

Currently, commercial CAD systems for lung nodule detection have only been approved for use as a second or concurrent reader [2]. In the setup of a second reader, the radiologist evaluates a study first without the help of CAD and then uses CAD to adapt the findings, adding or rejecting lesions from the original interpretation. In the setup as concurrent reader, the radiologist uses the CAD output *while* interpreting the study, accepting or rejecting CAD-detected nodules and adding nodules missed by CAD. Using CAD as a pre-screening reader is a potential new third application, in which the system automatically identifies normal cases that require no interaction with a radiologist, thus substantially reducing the workload of radiologists [3]. This is relevant because the majority of screenees do not have any lung nodules [4] and some DL-CAD algorithms have already shown equivalent or even superior performance compared to radiologists [5, 6].

At present there is little knowledge under which specific conditions CAD systems for lung nodule detection will save costs in a screening setting. The aim of this study is to gain insight into potential business cases for DL-CAD in a lung cancer screening setting in Western countries. We only focus on the workflow aspect of the business case for DL-CAD and therefore work under the simplified assumption that the CAD will only change workflow and will not change reporting accuracy.

## Methods

We used published data on efficiency gains in nodule detection by DL-CAD to calculate break-even points for various pricing models of commercial CAD systems. We performed the analysis in three representative Western countries, the USA, the UK, and Poland, in which lung cancer screening is implemented or currently considered [7–9].

A scoping review was performed to get an overview of reading time with and without DL-CAD assistance. The published studies were retrieved from the PubMed database from its inception to Oct 16, 2022. The search string consisted of the following keywords (Table S1): computed tomography, reading time, computer-aided detection, and lung nodule. Since we focused on the use of commercially available DL-CAD in a lung screening setting, articles were excluded if (1) contrast-enhanced CT was included, (2) non-deep learning-based CAD was evaluated, and (3) non-commercial DL-CAD was evaluated. The extracted data included the first author, publication year, country, normal or low dose CT, name of the commercial DL-CAD system, and reading time per CT scan with and without DL-CAD assistance. The reading time extracted from each study was pooled with random effect model.

Since no studies evaluated the reduction in reading time of DL-CAD as a pre-screening reader, we estimated it based on the proportion of normal CT scans in a screening setting. According to a recent review, 22–51% of participants (depending on the detection limit of size) in screening RCTs have a lung nodule detected at baseline [4]. We assumed that a DL-CAD would be able to exclude 80% of the nodule-free cases to allow the DL-CAD to be set to a very high sensitivity at a modest specificity just to be sure not to lose any potentially actionable nodules. Therefore DL-CAD as a pre-screening reader would reduce 39–62% of the workload of a radiologist and thus would save 39–62% reading time. We took the best-case and worst-case scenarios in which 62% and 39% of the reading time was saved, respectively.

Actual pricing for the DL-CAD system depends on country, pricing model, and local negotiations. While exact numbers are not publicly available, the National Institute for Health and Care Excellence (NICE) has published numbers for the UK in 2021 [10]. There are usually direct cost involved around installation and training, which were in the range of £8500–9000 (€9971–10,558, 1 GBP = 1.1731 EUR on average in 2022 [11]). In addition, there are yearly fees of £4000–14,800 (€4692–17,362) consisting of cost for hosting, monitoring, and support. Currently, three different pricing models are offered by various companies to pay for the actual use of the DL-CAD: a pay-per use model, a one-off perpetual software license, and a yearly subscription model. Data published by NICE suggests pay-per-use pricing of £5–7.5 (€5.9–8.8) and one-off pricing of £44,000 (€51,616). Yearly subscription costs usually vary according to the expected number of scans to be processed per year, estimated at €20,000 per year.

The payment to a radiologist per hour [12] is approximately £108 in the UK (€127, 1 GBP = 1.1731 EUR), $206 in the USA (€196, 1 USD = 0.9518 EUR [13]), and zł 211 in Poland (€45, 1 PLN = 0.2135 EUR [14]).

For these data, we calculated the break-even points at which using DL-CAD for nodule detection would start to become cost-saving using the three various pricing models, and the three reading paradigms. For the purpose of this study, we used the cost settings of the UK, USA, and Poland.

For the pay-per-use model, we calculated the cost per case for break-even. For the one-off perpetual software license and for the yearly subscription model we determined the break-even by calculating the minimum workload required to earn back the investment.

The cost (*C*) per case for break-even was calculated as *C* = S*Δ*t*, where *S* are the gross salary costs of a radiologist in euros per hour, and Δ*t* is the saved time in hours per CT scan with DL-CAD assistance. The minimum workload (*W*) in the number of assisting CT scans reading of a DL-CAD system was calculated as *W* = *P*/(S*Δ*t*), where *P* is the price (in euros) of a commercial DL-CAD system, *S* are the gross salary costs of a radiologist in euros per hour, and Δ*t* is the saved time in hours per case with DL-CAD assistance.

## Results

The identified studies and extracted key information about the reading time with and without DL-CAD are presented in Table 1. The pooled mean reading time without DL-CAD assistance was 162 (95% CI: 111–212) s. DL-CAD as a second reader increased the mean reading time by 33–41 s as compared to image reading without DL-CAD across studies. DL-CAD as a concurrent reader saved the mean reading time by 77 (95% CI: 47–107) seconds. Assuming best-case and worst-case scenarios that save 62% and 39% reading time, DL-CAD as a pre-screening reader would save the mean reading time by 100 (95% CI: 69–131) and 64 (95% CI: 43–83) seconds, respectively.

**Table 1:** Summary of the literature reported reading time for CT scans with and without DL-CAD assistance. *Reading time in mean (*± *SD) or median (IQR)*

Study	Country or region	CT modality	DL-CAD product	Reading time (s)		Time difference (s)	*P* value
				**No DL-CAD**	**DL-CAD**		
**DL-CAD as a second reader**							
Hsu (2021) [15]	Taiwan	Low dose CT	ClearReadCT (Riverain Technologies, Miamisburg, OH, USA)	156 ± 34	197 ± 46	41 [95% CI:39 to 44]	< 0.001
Vassallo (2019) [16]	Italy	CT	M5L lung CAD on-demand, INFN	296 ± 80	329 ± 83	33	< 0.05
**DL-CAD as a concurrent reader**							
Hempel (2022) [17]	The Netherlands	CT	Veye Chest v2.15.3, Aidence B.V., Amsterdam, NL)	Reader 1: 226.4 ± 113.2 Reader 2: 320.8 ± 164.2	Reader 1: 150.8 ± 74.2 Reader 2: 184.2 ± 125.3	Reader 1: − 75.6 Reader 2: − 136.6	< 0.001
Jacobs (2021) [18]	USA	Low dose CT	Veolity Lung CAD, version 1.5, MeVis Medical Solutions	160 (96–245)	86 (51–141)	− 64 (IQR: − 8 to − 137)	< 0.001
Hsu (2021) [15]	Taiwan	Low dose CT	ClearReadCT system (Riverain Technologies, Miamisburg, OH, USA)	156 ± 34	124 ± 25	− 32 [95% CI: − 30 to − 44]	< 0.001
Kozuka (2020) [19]	Japan	CT	InferRead CT Lung	186	168	− 18	Not provided
Lo (2018) [20]	USA	Low dose CT	ClearRead CT Vessel Suppression, Riverain Technologies	127.2	95.6	− 31.6 (95% CI: − 15.9 to − 47.4)	< 0.01
**Pooled**	–	–	–	162 (95% CI: 111–212)	118 (95% CI: 82–154)	77 (95% CI: 47–107)	–

**Fig. 1:**
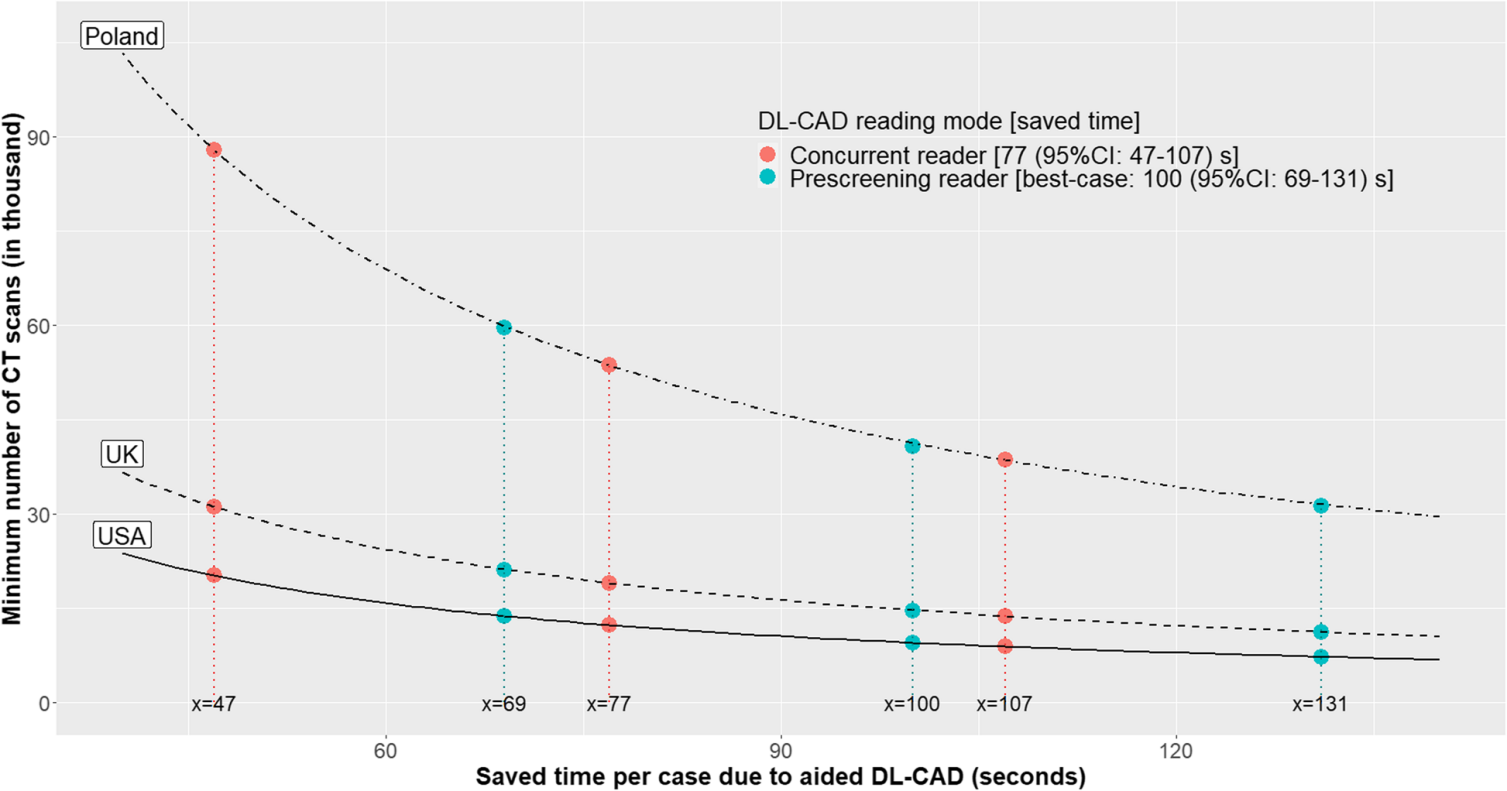
Minimum number of CT scans (as a function of saved reading time) for break-even using a DL-CAD system for nodule detection in the USA, UK, and Poland (one-off perpetual software license model)

The break-even pricing or minimum workload of DL-CAD depended on its reading mode and country setting. With the pay-per-use model, for concurrent reader, the break-even price was €4.2 (95% CI: 2.6–5.8) in the USA, €2.7 (95% CI: 1.7–3.8) in the UK, and €1.0 (95% CI: 0.6–1.3) in Poland. For pre-screening reader between the best- and worst-case scenario, the break-even price ranged from €3.5 to €5.4 in the USA, €2.3 to €3.5 in the UK, and €0.8 to €1.3 in Poland. With the one-off perpetual software license model, for concurrent reader, the minimum workload (CT scans in thousands) was 12.3 (95% CI: 8.9– 20.2) in the USA, 19.0 (95% CI: 13.7–31.1) in the UK, and 53.6 (95% CI: 38.6–87.9) in Poland. For pre-screening reader between the best- and worst-case scenarios, the minimum workload (CT scans in thousands) ranged from 9.4 to 14.9 in the USA, 14.5 to 23.0 in the UK, and 40.8 to 65.0 in Poland. With the yearly subscription model, for concurrent reader, the minimum workload (CT scans in thousands per year) was 4.8 (95% CI: 3.4–7.8) in the USA, 7.4 (95% CI: 5.3–12.1) in the UK, and 20.8 (95% CI: 15.0–34.0) in Poland. For pre-screening reader between the best- and worst-case scenarios, the minimum workload (CT scans in thousands per year) ranged from 3.6 to 5.8 in the USA, 5.6 to 8.9 in the UK, and 15.8 to 25.2 in Poland (Table 2). For the one-off perpetual software license model, the minimum number of CT scans required as a function of DL-CAD saved time by countries is visualized in Fig. 1. A Supplementary Excel file for calculations in various settings and DL-CAD prices is attached.

**Table 2:** Cost per case or minimum workload for break-even using a DL-CAD system for lung nodule detection in three salary cost settings of the USA, UK, and Poland

Pricing model	Price (€)	Cost per case for break-even (€)			Minimum workload (CT scans in thousands)		
		USA, €196/h	UK, €127/h	Poland, €45/h	US, €196/h	UK, €127/h	Poland, €45/h
**Concurrent reader, saved time (seconds) of 77 (95% CI: 47–107) per case**							
Pay-per-use model	5.9–8.8	4.2 (95% CI: 2.6–5.8)	2.7 (95% CI: 1.7–3.8)	1.0 (95% CI: 0.6–1.3)	–	–	–
One-off perpetual software license	51,616	–	–	–	12.3 (95% CI: 8.9–20.2)	19.0 (95% CI: 13.7–31.1)	53.6 (95% CI: 38.6–87.9)
Yearly subscription model^a^	20,000	–	–	–	4.8 (95% CI: 3.4–7.8)	7.4 (95% CI: 5.3–12.1)	20.8 (95% CI: 15.0–34.0)
**Pre-screening reader, best-case scenario: saved time (seconds) of 100 (95% CI: 69–131) per case**							
Pay-per-use model	5.9–8.8	5.4 (95% CI: 3.8–7.1)	3.5 (95% CI: 2.4–4.6)	1.3 (95% CI: 0.9–1.6)	–	–	–
One-off perpetual software license	51,616	–	–	–	9.4 (95% CI: 7.2–13.7)	14.5 (95% CI: 11.1–21.1)	40.8 (95% CI: 31.2–59.6)
Yearly subscription model^a^	20,000	–	–	–	3.6 (95% CI: 2.8–5.3)	5.6 (95% CI: 4.3–8.2)	15.8 (95% CI: 12.1–23.1)
**Pre-screening reader, worst-case scenario: saved time (seconds) of 64 (95% CI: 43–83) per case**							
Pay-per-use model	5.9–8.8	3.5 (95% CI: 2.3–4.5)	2.3 (95% CI: 1.5–2.9)	0.8 (95% CI: 0.5–1.0)	–	–	–
One-off perpetual software license	51,616	–	–	–	14.9 (95% CI: 11.4–21.8)	23.0 (95% CI: 17.6–33.6)	65.0 (95% CI: 49.7–94.9)
Yearly subscription model^a^	20,000	–	–	–	5.8 (95% CI: 4.4–8.4)	8.9 (95% CI: 6.8–13.0)	25.2 (95% CI: 19.3–36.8)

*USA* the United States, *UK* the United Kingdom, *95% CI* 95% confidence interval

^a^Data are shown for per year

## Discussion

In this study evaluating the pricing and cost-saving potential for a DL-CAD system in lung nodule detection in a screening setting, using a straightforward model, we found that a break-even use of a DL-CAD will require the per-case cost substantially below €6 or it being used in a high-workload environment, especially in a country where the price of a radiologist is cheap. DL-CAD as a pre-screening reader shows the largest potential to be cost-saving.

DL-CAD as concurrent reader has been approved for clinical use and is commercially available. Based on our scoping review, DL-CAD as a concurrent reader saves reading time for a radiologist by more than one minute per case. Furthermore, it is shown that DL-CAD as a concurrent reader significantly improves the performance of nodule detection (sensitivity increased from 64 to 80%, *p* < 0.001) [15]. Therefore, the utility of DL-CAD as a concurrent reader will be preferable for improvement of the workflow efficiency when lung cancer screening is implemented. By monetizing the saved time, our study showed that with the pay-per-use pricing model of DL-CAD products, a break-even use of DL-CAD as concurrent reader will require a much lower per-case price of €1.0–4.2 than the current price of €5.9–8.8, especially in a country like Poland. With the pricing model of the one-off perpetual software license, the minimum workload required is extremely high to make DL-CAD cost-saving. It means a screening institute with 1000 CT cases per year must use it for at least 12 years in the USA, 19 years in the UK, and 54 years in Poland to make it break-even. With the yearly subscription model, the minimum workload must be nearly 21 thousand CT scans per year in Poland to earn back the investment. The workload could be much higher than the practice in a country like Poland. This informs policy-makers on the minimal population coverage of a screening center, but for a low-salary and small population-size country the current high price of DL-CAD would deter the purchase and usage.

DL-CAD as a second reader increases the reading time for a radiologist as expected, since the radiologist first performs an unaided interpretation and then reviews the outputs of DL-CAD. Consequently, our study showed that DL-CAD as a second reader in lung cancer screening is not cost-saving. However, it should be noted that the use of DL-CAD as a second reader can identify missed pulmonary nodules, some of which may be clinically significant [21, 22]. This potential benefit of DL-CAD as a second reader is out of the scope of the current study.

Since a majority of screenees joining in a population lung cancer screening have a negative CT scan (no nodule or micro-nodules below detection limit) [4], DL-CAD as pre-screening reader ruling out those scans has the largest potential to be cost-saving. As shown in our study, compared to the concurrent reading paradigm, DL-CAD as pre-screening reader requires a higher per-case cost and lower workload to reach break-even. This is because DL-CAD as pre-screening reader rules out a large proportion of negative CT scans and thus will save much time for a radiologist. It is noted that this merit comes along with a drawback. When DL-CAD is trained for only lung nodule detection, the use of DL-CAD as pre-screening reader might miss other abnormalities in the lung or chest. This suggests that DL-CAD aiming for ruling out negative chest scans should be used as a pre-screening reader to largely avoid overlooked abnormalities in the chest.

We found that the lower the salary of a radiologist, the higher the required workload. In a relatively low-salary country like Poland, the number of CT scans must be at least 53.6 thousand for DL-CAD as a concurrent reader to be cost-saving. That means DL-CAD must be cheaper for a wide-spread and sustainable use.

To our knowledge, this is the first study to evaluate the pricing and cost-saving potential for a commercial DL-CAD nodule detection system. The results are informative for pricing of DL-CAD products vendors and users. To adapt our findings to local settings, we developed an Excel file for calculations with different assumptions in the Supplemental file. However, there are some limitations in this study. First, we applied the reported mean reading time saved to estimate the minimum number of CT scans, while it is well known that reading time largely depends on the experience of readers. As shown in a study that DL-CAD could help junior readers save more time than senior readers [15]. However, most of the identified studies included both junior and senior readers. So the impact on our results was attenuated. Second, the price-setting of DL-CAD in reading CT scans was not standardized., The price can be dependent on the country, the number of users, the number of installations or the number of CT analyses [1]. We applied the cost of DL-CAD reported in the UK, whereas this may vary among different countries and vendors. However, a different price is easy to adapt in our concise model. Third, variation existed in the definition of reading time in the identified studies. Some studies reported the time for nodule detection and measurement while others reported time for completing a CT study including writing CT report and/or waiting for loading a case. More studies are warranted to evaluate the impact of DL-CAD on the time for the whole workflow and on cost-efficiency. Last, we only considered the costs involved in DL-CAD, as the costs related to downstream testing of nodules and cancer diagnosis were beyond the aim of this study.

In conclusion, the current prices of DL-CAD systems for nodule detection in lung cancer screening are far beyond cost-saving for a screening institute, especially in low-salary countries. DL-CAD as a pre-screening reader shows the largest potential to be cost-saving, but just in case of a huge workload of CT scans. DL-CAD as second reader in lung cancer screening is not cost-saving. DL-CAD products must be cheaper for a wide-spread and sustainable use.

### Supplementary Information

**Additional file 1. Table S1.** Search query of the scoping review for reading time of CT scan with and without DL-CAD assistance.

**Additional file 2.** Calculation pricing and workload.

## Data Availability

All data generated or analyzed during this study are included in this published article.

## Abbreviations

DL-CAD: Deep learning computer-aided detection
CT: Computed tomography
