# Supplementary material for: Pricing and cost-saving potential for deep-learning computer-aided lung nodule detection software in CT lung cancer screening
Source: Insights Imaging. 2023 Nov 27;14:208. doi: 10.1186/s13244-023-01561-z (PMC10682324; doi:10.1186/s13244-023-01561-z)
Supplement: Supplementary file 1 — Additional file 1. Table S1. Search query of the scoping review for reading time of CT scan with and without DL-CAD assistance. [file 13244_2023_1561_MOESM1_ESM.docx]

**Table S1** Search query of the scoping review for reading time of CT scan with and without DL-CAD assistance

| **Boolean operator** | **String** |
| --- | --- |
|  | (((((Tomography, X-Ray Computed[MeSH Terms])) OR (computed tomography[Title/Abstract])) |
| AND | (((reading[Title/Abstract]) OR (interpretation[Title/Abstract])) AND (time[Title/Abstract]))) |
| AND | ((computer-aided detection[Title/Abstract]) OR (assisted[Title/Abstract]))) |
| AND | (((lung[Title/Abstract]) OR (pulmonary[Title/Abstract])) AND (nodule*[Title/Abstract])) |
